# Noise exposure and hypertension: investigation of a silent relationship

**DOI:** 10.1186/s12889-015-1671-z

**Published:** 2015-04-03

**Authors:** Tatiana Cristina Fernandes de Souza, André Reynaldo Santos Périssé, Marisa Moura

**Affiliations:** Centro de Estudos da Saúde do Trabalhador e Ecologia Humana, Escola Nacional de Saúde Pública Sergio Arouca, Fundação Oswaldo Cruz (CESTEH/ENSP/Fiocruz), Rua Leopoldo Bulhões 1480, Manguinhos, Rio de Janeiro/RJ Brazil; Departamento de Ciências Biológicas, Escola Nacional de Saúde Pública Sergio Arouca, Fundação Oswaldo Cruz (DCB/ENSP/Fiocruz), Rua Leopoldo Bulhões 1480, Manguinhos, Rio de Janeiro/RJ Brazil

**Keywords:** Occupational Noise, Hypertension, High Blood Pressure, Occupational Exposure, Occupational Health

## Abstract

**Background:**

Noise is an important occupational hazard worldwide and hypertension a well-known risk factor for cardiovascular disease, which is currently the greatest cause of disability retirement worldwide. The association between noise exposure and auditory effects is well documented in the biomedical literature, but the same is not true about exposure to different levels of noise and extra-auditory effects. It has been shown that noise exposure levels to be considered for non-auditory effects may not be the same as in the case of auditory effects. The frequent presence of noise in workplace environments, the high prevalence of hypertension worldwide, the biological plausibility of the association between noise exposure and high blood pressure and the need for more studies investigating the non-auditory effects of exposures to less than 85 dB(A), were the reasons that led us to develop this study. We aimed at investigating the hypothesis that exposure to different levels of noise is associated with hypertension.

**Methods:**

We used a cross-sectional design to study the association between occupational noise exposure (≤75, 75–85, and ≥ 85 dB(A)) and hypertension (use of anti-hypertensive medication and/or blood pressure of ≥140/90 mmHg) in 1,729 petrochemical workers at Rio de Janeiro, Brazil. Data were collected from obligatory annual health evaluation records and from environmental measurements of noise and heat levels. We used logistical regression analysis to study the association while controlling for key confounding variables, such as smoking and body mass index.

**Results:**

Using the ≤75 dB(A) as reference category, noise exposure was independently associated to hypertension both at the 75–85 dB(A) (OR 1.56; 95% CI 1.13–2.17) and the ≥85 dB(A) levels (OR 1.58; 95% CI 1.10–2.26). Age, gender and body mass index were also independently associated to high blood pressure.

**Conclusion:**

Herein, we were able to demonstrate that noise exposure is independently associated to hypertension. Our results are consistent with other studies that used similar methodology and enabled us to verify the occurrence of non-auditory effects in workers exposed to noise levels considered safe for auditory effects.

## Background

Hearing loss is the most investigated and accepted health effect related to noise exposure [1-4]. However, other health effects such as digestive and behavioral disorders [5], sleep disturbances [6], changes in the serum cortisol levels [7], cardiovascular diseases [8] and a higher incidence of occupational accidents [9,10] may also be associated to noise exposure.

Studies of the association between noise and outcomes not related to hearing disease are usually based on analogies to the hearing effects of noise, and the maximum exposure limits for hearing effects (85 dB(A)) is usually also considered for non-hearing outcomes [11]. However, there are evidences that lower level exposure to noise is associated to other health effects. Berglung et al. [12] reviewed the biomedical evidence of the association between noise exposure and non-hearing effect and were able to show a higher level of stress among individuals exposed to 55 dB(A) and cardiovascular disturbances in individuals exposed to more than 65 dBA.

Cardiovascular diseases was one of the leading causes of death and years of life lost due to premature mortality (YLLs) in the 2010 report of global burden of disease [13]. High blood pressure is highly associated to cardiovascular disease and its high prevalence worldwide makes it one of the three leading risk factor for global disease burden [14]. The World Health Organization (WHO) estimated that, in 2008, 30% of the adults in the WHO European Region and 23% in the WHO Region of the Americas had high blood pressure [15]. Almost 9.5 million deaths each year, or 16.5% of all deaths worldwide can be attributed to high blood pressure [14]. According to the Brazilian Hypertension Society, high blood pressure affects, on average, 25% of the Brazilian population [16].

The hypothesis that exposure to excessive noise could be associated to high blood pressure was described by Babisch [8] who showed that the association could be explained by the biochemical changes related to the mechanisms of stress. Briefly, an increase in the level of chemical substances like cortisol, adrenaline and noradrenaline in response to the stress caused by noise could result in peripheral vasoconstriction, increased heart rate, and increased arterial blood pressure [11,17-19]. In workplace environments, other stress-generated situations sharing with noise the same physiopathological mechanism may exist, which may contribute to the high prevalence and incidence of hypertension in these locations [20-23].

The frequent presence of noise in workplace environments, the high prevalence of hypertension worldwide, the biological plausibility of the association between noise exposure and high blood pressure and the need for more studies investigating the non-auditory effects of exposures to less than 85 dB(A), were the reasons that led us to develop this study.

## Methods

### Study design and population

To address the main question of the association between noise exposure and high blood pressure, we performed a cross-sectional study design of existent secondary health and noise exposure data from 2007 related to workers of a petrochemical and gas refinery in Rio de Janeiro, Brazil.

The studied unit has two types of work force: contractors (workers paid by the government) and sub-contractors (workers paid by outsourced corporations). The two workforces are subject to different labor legislations and all outsourced corporations are required to perform pre-admission and annual health examination in every worker. Subcontracted labor force was working on the plant maintenance and expansion, while government workers were more involved in administrative tasks in the plant. Our sample framework was based only on workers from outsourced corporations (sub-contractor workers) since data was available only to them. We used the 6-month timeframe in order to guarantee a minimum period of noise exposure before the diagnosis of hypertension. The total number of subcontractor workers was 3,023 individuals, but our study was restricted to the 1,729 individuals working at the petrochemical industry for at least 6 months and submitted to annual physical exam in 2007.

### Health, socioeconomic and environmental data

To elaborate our dataset we collected information from the electronic clinical forms of workers submitted to the annual mandatory health evaluation.

The variable age was stratified in five-year intervals. Civil status was dichotomized in married and single/divorced individuals, the only available categories. Formal education was reclassified in completed or incomplete elementary school, middle school, high school and college. Physical activity, tobacco use and alcohol consumption were defined according to the clinical form. Thus, regular physical activity included any activity for at least three times a week and a minimum duration of 20 minutes. Regular alcohol consumption was attributed to individuals informing daily alcohol ingestion, occasional consumption to individuals informing alcohol use only on weekends, and individuals informing no alcohol consumption were classified as no consumption. No data were available about the amount, type of alcoholic beverage and history of alcohol consumption. Alcohol consumption data was further categorized in consumption and no consumption, which encompassed those that consumed alcoholic beverages both occasionally and regularly. Body Mass Index (BMI) was calculated from the values of weight and height and categorized as normal (underweight - <18.50 Kg/m^2^ and normal – 18.50-24.99 Kg/m^2^), overweight (25.00-29.99 Kg/m^2^) and obese (≥30.00 Kg/m^2^) according to the WHO criteria [24]. Heating exposure was classified as exposed, when an individual was found to be exposed to values equal to or higher than 30 WBGTI (Wet Bulb Globe Thermometer Index) by the time of the measurement, according to a methodology recommended by the Fundacentro [25].

Traditional socioeconomic variables were not available in the forms from the annual mandatory health evaluation. Therefore, we used all available information that could be linked to the social condition of the workers. Hence, presence of running water, regular trash collection, sewage network and electrical power were used to create a composite indicator in which each of the four variables was classified as absent (0) or present (1), assuming equal importance to all variables. The socioeconomic indicator was classified as higher if all four variables were present and lower if at least one variable was absent.

There were several sectors distributed along the different subcontracted firms and with different levels of exposure to noise, and we were unable to obtain a correct estimate of the location of the workers per sector. Therefore, we opted for grouping the workers by the type of work in the industry as in “industrial maintenance”, “cleaning and building maintenance”, “civil works” and “others” (meals, transportation and security).

### Definition of exposure and outcome

The outcome (high blood pressure) was defined as systolic pressure equal to or higher than 140 mmHg and/or a diastolic pressure equal to or higher than 90 mmHg [26]. Any worker reporting the use of anti-hypertensive medication was also classified as hypertensive, regardless of the measured pressure level. Blood pressure was measured by the attendant physician once at the beginning of the medical consultation, always on the left arm of a patient seated. In cases in which the blood pressure measurement was above the cut-off limits, a second measurement was performed at the end of the consultation. In these situations, only the second measurement was reported in the medical records.

Exposure to noise was measured at Homogeneous Exposure Groups (HEG) by using a digital audio dosimeter affixed next to their aural point to a randomly selected worker within the HEG. The measurement was done at a single moment in cases of continuous exposure to noise and during at least 75% of a workday of 8 hours in cases of intermittent exposure to noise, according to the Fundacentro methodology [27]. We categorized noise exposure in three levels: ≤75 dB(A), from 75 to 85 dB(A), and ≥85 dB(A). We based our decision to use the three level categorization on some studies that also considered lower levels for noise exposure [12,22,28].

### Statistical analysis

Since the available dataset was not created to be used in a research project, not all-important variables were readily available to be used in the statistical analyzes. We decided to analyze confounding and interaction terms on any available variable with some indication in the biomedical literature of association to either noise exposure or high blood pressure. Therefore, age, gender, marital status, education, socioeconomic condition, physical activity, tobacco use, alcohol consumption, body mass index (BMI), type of service and heat exposure were the co-variables used in our analysis.

We began our data analysis by describing the frequency distribution of all variables according to the level of noise exposure. Thereafter, we performed a bivariate analysis for the association between all co-variables and both the exposure and the outcome variables in order to identify possible confounding variables for the studied association. All variables were categorized and a Chi-squared test used to test the associations. Any variable associated to the outcome with a p-value of at least <0.15 was considered to be included in the final multivariate model.

First order interactions terms between noise and age, gender, socioeconomic condition, physical activity, tobacco use, consumption of alcohol, body mass index and exposure to heat were individually tested and considered for the inclusion in the final multivariate model. Therefore, the baseline multivariate model was composed by all variables with a p-value of less than 0.15 in the exploratory analysis and statistically significant interaction terms. A logistic regression model with backward Wald elimination was used to model the independent association between noise exposure and high blood pressure, while considering other possible confounding variables. We present the final multivariate model only with variables statistically associated with high blood pressure since addition of non-significant variables did not add to the fitness of the final logistic model. The final odds ratio represents the relative odds of presence of high blood pressure. Statistical analysis was performed with the statistical package SPSS (version 17; SPSS *Inc. software products*).

The study was approved by the Ethics Committee at the National School of Public Health (CEP/ENSP). We analyzed a secondary dataset with no identifiers and the study was granted with a consent form waiver by the CEP/ENSP.

## Results

Data from 1,729 workers were included in our analysis. The prevalence of high blood pressure among sampled workers was approximately 25% (430/1729) (Table 1). Fifty-five out of the 430 (12.7%) individuals classified as hypertensive were already under medication. Twenty-two percent of the individuals were exposed to levels ≤ 75 dB(A) (388/1,729), 50% to levels between 75 and 85 dB(A) (871/1,729), and 27% were exposed to levels ≥ 85 dB(A) (470/1,729). Most of the workers were male (1,585/92%), younger than 40 years-old (956/55%) and single (919/53%). Men and married workers had a higher probability to be exposed to the highest level of noise (p = 0.00 and p-0.01, respectively). Fewer workers had college-level education (124/7%) and workers with higher formal education were more likely to be exposed to lower levels of noise (p = 0.00). Regular physical activity was reported by 21% (n = 367) of the individuals and 58% (n = 1,011) of the workers were overweight or obese. Workers were mostly non-smoker (1,346/78%) and not a regular consumer of alcohol (n = 1,078/62%). Most of the sampled population was involved in industrial maintenance (610/35%) and civil construction services (615/35.6%). Approximately 90% of them (n = 1,526) of them were not exposed to heat, according to the used exposure criteria. Workers in industrial maintenance were more likely to be in the higher level of noise exposure group (p = 0.00) as well as workers exposed to heat (p = 0.00).

**Table 1:** **General characteristic of the sample by levels of noise exposure**

**Variable**	**≤75 dB(A)**	**75 – 85 dB(A)**	**≥85 dB(A)**	**p-value**
	**n (%)**	**n (%)**	**n (%)**	
	**n = 388**	**n = 871**	**n = 470**	
**Age (years)**				
<30	83 (23,2)	186 (52,0)	89 (24,9)	0.03
30-34	80 (26,1)	145 (47,4)	81 (26,5)	
35-39	56 (19,2)	158 (54,1)	78 (26,7)	
40-44	42 (15,4)	147 (53,8)	84 (30,8)	
45-49	47 (21,8)	107 (49,5)	62 (28,7)	
≥50	80 (28,2)	128 (45,1)	76 (26,8)	
**Gender**				
Female	82 (56,9)	60 (41,7)	2 (1,4)	0.00
Male	306 (19,3)	811 (51,2)	468 (29,5)	
**Marital status**				
Single	212 (23,1)	485 (52,8)	222 (24,2)	0.01
Married	176 (21,7)	386 (47,7)	248 (30,6)	
**Education**				
Up to incomplete elementary school	11 (13,6)	45 (55,6)	25 (30,9)	0.00
Complete elementary school	24 (18,0)	62 (46,6)	47 (35,3)	
Incomplete middle school	40 (11,4)	202 (57,7)	108 (30,9)	
Complete middle school	45 (14,9)	173 (57,3)	84 (27,8)	
Incomplete high school	39 (19,3)	96 (47,5)	67 (33,2)	
Complete high school	157 (29,2)	252 (46,9)	128 (23,8)	
College/university (complete or not)	72 (58,1)	41 (33,1)	11 (8,9)	
Socioeconomic condition*				
Lower	71 (16,8)	242 (57,2)	110 (26,0)	0.00
Higher	317 (24,3)	629 (48,2)	360 (27,6)	
**Physical activity**				
No	231 (22,5)	514 (50,1)	281 (27,4)	0.44
Irregular	66 (19,6)	171 (50,9)	99 (29,5)	
Regular	91 (24,8)	186 (50,7)	90 (24,5)	
**Tobacco use**				
Non-smoker	301 (22,4)	698 (51,9)	347 (25,8)	0.03
Smoker	87 (22,7)	173 (45,2)	123 (32,1)	
**Alcohol consumption**				
No consumption	252 (23,4)	556 (51,6)	270 (25,0)	0.03
Regular	136 (20,9)	315 (48,4)	200 (30,7)	
**Body Mass Index**				
Normal	167 (23,3)	373 (51,9)	178 (24,8)	0.04
Overweight	148 (19,9)	380 (51,2)	214 (28,8)	
Obese	73 (27,1)	118 (43,9)	78 (29,0)	
**Type of Service**				
Industrial maintenance	169 (27,7)	180 (29,5)	261 (42,8)	0.00
Civil Works	135 (22,0)	329 (53,5)	151 (24,6)	
Building cleaning	33 (9,4)	261 (74,1)	58 (16,5)	
Others	51 (33,6)	101 (66,4)	0 (0)	
**Heat**				
Non-exposed	385 (25,2)	769 (50,4)	372 (24,4)	0.00
Exposed	3 (1,5)	102 (50,2)	98 (48,3)	
**Arterial Hypertension**				
Normotensive	312 (24,0)	648 (49,9)	339 (26,1)	0.02
Hypertensive	76 (17,7)	223 (51,9)	131 (30,5)	

*Socioeconomic condition: Existent - presence of piped water + regular garbage collection + sewage system + electricity; absence: inexistence of at least one of the above factors.

Prevalence of hypertension was higher among older workers, especially after age 50 (n = 134/47.2%) (Table 2). Prevalence of high blood pressure was also higher among male (n = 413/26.1%) and married workers (n = 249/30.7%). Hypertension was more likely associated with no regular physical activity (n = 276/26.9%), tobacco use (n = 122/31.9%), alcohol consumption (n = 191/29.3%) and obesity (n = 121/45.0%).

**Table 2:** **Association between sociodemographic and environmental variables and blood pressure level**

**Variable**	**Normotensive**	**Hypertensive**	**p-value**
	**n (%)**	**n (%)**	
	**n = 1299**	**n = 430**	
**Age (years)**			
<30	332 (92,7)	26 (7,3)	0,00
30-34	260 (85,0)	46 (15,0)	
35-39	236 (80,8)	56 (19,2)	
40-44	186 (68,1)	87 (31,9)	
45-49	135 (62,5)	81 (37,5)	
≥50	150 (52,8)	134 (47,2)	
**Gender**			
Female	127 (88,2)	17 (11,8)	0,00
Male	1172 (73,9)	413 (26,1)	
**Marital status**			
Single	738 (80,3)	181 (19,7)	0,00
Married	561 (69,3)	249 (30,7)	
**Education**			
Up to incomplete elementary school	56 (69,1)	25 (30,9)	0,07
Complete elementary school	96 (72,2)	37 (27,8)	
Incomplete middle school	256 (73,1)	94 (26,9)	
Complete middle school	239 (79,1)	63 (20,9)	
Incomplete high school	157 (77,7)	45 (22,3)	
Complete high school	392 (73,0)	145 (27,0)	
College/university (complete or not)	103 (83,1)	21 (16,9)	
Socioeconomic condition*			
Lower	331 (78,3)	92 (21,7)	0,09
Higher	968 (74,1)	338 (25,9)	
**Physical activity**			
No	750 (73,1)	276 (26,9)	0,04
Irregular	257 (76,5)	79 (23,5)	
Regular	292 (79,6)	75 (20,4)	
**Tobacco use**			
Non-smoker	1038 (77,1)	308 (22,9)	0,00
Smoker	261 (68,1)	122 (31,9)	
**Alcohol consumption**			
No consumption	839 (77,8)	239 (22,2)	0,00
Regular	460 (70,7)	191 (29,3)	
**Body Mass Index**			
Normal	619 (86,2)	99 (13,8)	0,00
Overweight	532 (71,7)	210 (28,3)	
Obese	148 (55,0)	121 (45,0)	
**Type of Service**			
Industrial maintenance	464 (76,1)	146 (23,9)	0,29
Civil Works	453 (73,7)	162 (26,3)	
Building cleaning	274 (77,8)	78 (22,2)	
Others	108 (71,1)	44 (28,9)	
**Heat**			
Non-exposed	1154 (75,6)	372 (24,4)	0,19
Exposed	145 (71,4)	58 (28,6)	

*Socioeconomic condition: Existent - presence of piped water + regular garbage collection + sewage system + electricity; absence: inexistence of at least one of the above factors.

Categorized age, gender, marital status, education, socioeconomic status, physical activity, use of tobacco, alcohol consumption and BMI were associated to the outcome (blood pressure level) and were considered for inclusion in the final multivariate model as possible confounding variables along with noise exposure (exposure variable) and the first order interaction term gender*noise. However, only age, gender, BMI and noise exposure were independently associated to arterial hypertension (Table 3). The positive association between noise exposure and high blood pressure was found at two categories of noise exposure when compared to a reference category of ≤ 75 dB(A), 75–85 dB(A) (OR 1.56; IC95% 1.13 – 2.17 ) and ≥ 85 dB(A) (OR 1.58; IC95% 1.10 – 2.26).

**Table 3:** **Odds ratio and adjusted odds ratio for arterial hypertension among those exposed to noise**

**Variable**	**Non-adjusted Odds Ratio**	**IC (95%)**	**Adjusted Odds Ratio**	**IC (95%)**
**Age (years)**				
<30	1,00 (Ref.)	-	1,00 (Ref.)	-
30-34	2,26	1,36 – 3,75	1,92	1,14 – 3,23
35-39	3,03	1,85 – 4,97	2,57	1,55 – 4,26
40-44	6,00	3,72 – 9,59	4,87	3,00 – 7,89
45-49	7,66	4,71 – 12,44	6,24	3,80 – 10,26
> = 50	11,41	7,18 – 18,10	10,16	6,33 – 16,31
**Gender**				
Female	1,00 (Ref.)	-	1,00 (Ref.)	-
Male	2,63	1,57 – 4,42	1,88	1,08 – 3,29
**Body Mass Index**				
Normal	1,00 (Ref.)	-	1,00 (Ref.)	-
Overweight	2,46	1,89 – 3,22	2,12	1,60 – 2,81
Obesity	5,11	3,71 – 7,04	4,46	3,16 – 6,29
Noise*				
≤75 dB(A)	1,00 (Ref.)	-	1,00 (Ref.)	-
75 – 85 dB(A)	1,41	1,05 – 1,89	1,56	1,13 – 2,17
≥85 dB(A)	1,58	1,15 – 2,19	1,58	1,10 – 2,26

*Controlled for age, gender and body mass index.

## Discussion

### Overview

More than 80% of workers with high blood pressure in our sample were exposed to noise levels above 75 dB(A), approximately 52% to levels between 75–85 dB(A). In our final multivariate model, we were able to show that the exposure variable noise level and the co-variables age, sex and BMI were independently associated to high blood pressure. It is important to note that the same co-variables are established risk factors for high blood pressure in the biomedical literature [29-33]. Workers exposed to noise levels ≥ 85 dB(A) and those exposure to levels varying from 75–85 dB(A) presented a 60% higher probability of presenting high blood pressure when compared to workers exposed to noise levels ≤ 75 dB(A), after controlling for age, gender and BMI.

High blood pressure, just like any other non-communicable diseases, is multifactorial in its causes and each separated risk factor has its own level of association to the blood pressure. In our final model, noise exposure association to blood pressure is weaker than the association between the other variables and the outcome. However, if the association is indeed casual and the prevalence of occupational noise exposure is high, such as the case of several types of industries, then the fraction of high blood pressure that could be attributable to noise exposure can be substantial.

### Literature review of non-hearing effects of noise exposure

Our results are consistent with other studies that employed similar methodologies but different definitions for exposure and outcome. Some studies have observed the increase in blood pressure measures on workers exposed to noise but they have failed in characterizing such workers as hypertensive. Chang et al. [22] showed that both systolic and diastolic blood pressure were significantly higher among workers exposed to noise levels ≥ 85 dB(A). Powazka et al. [34] observed an average increase in the systolic blood pressure of 5 mmHg among workers at a steel factory exposed to 89 dB(A). Tomei et al. [35] performed a meta-analysis to investigate the association between cardiovascular abnormalities and chronic occupational exposure to noise. They were able to show a statistically significant increase in the systolic and diastolic blood pressures only among workers exposed to an average noise level of 92 dB(A). However, few authors have used a formal definition of high blood pressure when studying exposure to occupational noise. Narlawar et al. [36] described a higher prevalence of high blood pressure among 770 workers exposed to noise levels ≥ 98 dB(A) at an iron and steel company in India. Souto Souza et al. [37] observed that the exposure to occupational noise to levels of 85 dB(A) or more for more than 10 years was associated to high blood pressure among workers at an oil industry in Brazil, even after adjusting for age, educational level and obesity. The same association was not significant when time of exposure was not taken into account. Interestingly, Inoue et al. [38] studied 242 Japanese workers exposed to high noise levels and showed an inverse association between high blood pressure and noise level at workplace.

The absence of adequate limits for non-hearing effects of noise exposure may result in the occurrence of health effects possibly associated to harmful exposures, but not identified. The importance of investigations of non-hearing effects associated to lower levels of noise exposures is supported by the results of the few studies that used 75 dB(A) as the cut-off level. Gitanjali and Ananth (2004) were able to show abnormalities during the sleep phases for exposures to lower levels of noise, while Chang et al. (2003) showed transitory changes to blood pressure for similar noise exposure levels [28,39]. Chang et al. [40] studied workers from a screw factory in Taiwan and also showed a significant association between noise exposure to ≥ 70 dB(A) and ≥ 80 dB(A) and high blood pressure. We have decided to investigate the association between noise exposure and high blood pressure at three different levels, and we have demonstrated a higher prevalence of hypertension even at exposure levels considered safe by current legislations (≤85 dB(A)).

### Limitations

Herein, we used a cross sectional design to address the main question of association between noise exposure and high blood pressure and this type of study design does not allow us to guarantee that exposure precedes the outcome, a condition to confirm a cause-effect association. However, it is unlikely that workers that initially had high blood pressure have been intentionally allocated to workplaces presenting higher levels of noise exposure. Besides, health effects of noise exposure may be time-dependent as demonstrated by Souto Souza et al. [37], and the cross sectional design did not allow us to consider time as a variable in our analysis. However, as oppose to their work, we were able to show an association even with no information about time of exposure.

Completeness and quality of data was one of the main limitations in our study. Data were collected as part of the annual mandatory health evaluation required by law in Brazil and no regular quality control measures required by a scientific research was in place during data collection. This may have influence not only the availability of the required variables but also the accuracy of some important information. Workers were classified as hypertensive during their routine annual mandatory health evaluations based on two blood pressure measures taken at one single occasion. Such approach may have created a selection bias and misclassified healthy individuals into hypertensive workers, leading to overdiagnosis and overmedication. In addition, some variables historically associated to high blood pressure, such as socioeconomic level [30], that could confound the studied association had not been collected as part of the annual evaluation and were unavailable to be tested. However, indicators of socioeconomic conditions such as sewage and trash collection and presence of piped water at home were available in the dataset, and we were able to create a composited indicator with such primary variables and use it as a proxy of socioeconomic status among the workers at the oil industry. The absence of association between the new indicator and high blood pressure may be real, but it may also indicate that the created indicator was not able to capture the real socioeconomic status among workers in our study. The use of personal protection equipment (PPE) such hearing protection may also determine different levels of exposure to noise [41]. Accurate information about the use of PPE was not available in the dataset.

### Public health relevance

It has been shown that subcontractor workers are more exposed to inadequate work conditions [42,43]. Therefore, the high frequency of noise exposure at workplace and the high prevalence of high blood pressure described in our population confirm the importance of our study. Hypertensive workers may have the exposure to lower levels of occupational noise associated to the development or worsening of high blood pressure hypertension. However, high blood pressure may not be considered in health evaluations since noise exposure is situated in levels considered safe according to current legislation. Therefore, it is important that noise exposure limits are established aiming at preventing both hearing and non-hearing health effects.

## Conclusion

The main idea of our study was to give elements to support the hypothesis of an association between noise exposure and high blood pressure. However, there is no indication why our results could not be applied to locals with similar noise exposure. Even with all limitations, we believe that our results are important as one further data presenting an association between noise exposure and high blood pressure due to the uncertainty still surrounding the issue in the biomedical literature. The association between noise exposure and auditory effects is well established in the environmental epidemiology, but the same is not true with non-auditory effects. Moreover, it has been shown that the noise exposure levels to be considered in the case of non-auditory effects may not be the same as in the case of auditory effects. Our results may contribute to emphasize the importance of searching for adverse health effects on exposure levels considered safe and alert decision makers and regulatory agencies about the importance of establishing more accurate noise exposure limits so that the health of workers is not compromise. The study may also serve to increase workers’ awareness about the occupational effects on health.

## Abbreviations

BMI: Body Mass Index
CEP/ENSP: Ethics Committee at the National School of Public Health
HEG: Homogeneous Exposure Groups
OR: Odds ratio
PPE: Personal protection equipment
WBGTI: Wet Bulb Globe Thermometer Index
WHO: World Health Organization
YLLs: Years of life lost due to premature mortality
